# Assessment and Characterization of Induced Alloantigen-Specific Regulatory T Cells Obtained by the Inhibition of CDK8/19 with the AS2863619 Compound

**DOI:** 10.3390/ijms262210957

**Published:** 2025-11-12

**Authors:** Aleksey Bulygin, Marina Fisher, Vasily Kurilin, Saleh Alrhmoun, Roman Perik-Zavodskii, Olga Perik-Zavodskaia, Marina Volynets, Nadezhda Shkaruba, Irina Obleukhova, Julia Khantakova, Elena Golikova, Alexandr Silkov, Sergey Sennikov

**Affiliations:** 1 Laboratory of Molecular Immunology, Federal State Budgetary Scientific Institution Research Institute of Fundamental and Clinical Immunology, 630099 Novosibirsk, Russia; aleksej.bulygin95@mail.ru (A.B.); msolshanova@gmail.com (M.F.); vkurilin@niikim.ru (V.K.); saleh.alrhmoun1@gmail.com (S.A.); zavodskii.1448@gmail.com (R.P.-Z.); mrsmarinavolynets@gmail.com (M.V.); sen-nadezhda@yandex.ru (N.S.); obleukhova.irina@yandex.ru (I.O.); khantakovain@bionet.nsc.ru (J.K.);; 2 Federal State Autonomous Educational Institution of Higher Education I.M. Sechenov, First Moscow State Medical University of the Ministry of Health of the Russian Federation, 119435 Moscow, Russia; elenagolikova329@gmail.com

**Keywords:** regulatory T cells, ag-Tregs, mouse, AS2863619, NanoString, flow cytometry, cytokines, migration assay

## Abstract

Foxp3+ regulatory T (Treg) cells play a pivotal role in inducing immune tolerance. The expression of Foxp3 in Treg cells depends on the stability of transcription factors that are directly linked to the molecular interplay between Stat5a and cyclin-dependent kinase CDK8/19. In this study, dendritic cells obtained from C57BL/6 male mice were co-cultured with CD4^+^ splenocytes obtained from Balb/c male mice to obtain alloantigen-specific CD4^+^ T cells. Next, these alloantigen-specific CD4^+^ T cells were cultured with the addition of the CDK8/19 inhibitor AS2863619 compound, TGF-β1, and IL-2 to induce their transdifferentiation into alloantigen-specific CD4^+^ Foxp3+ Treg cells. The efficacy of this cocktail in promoting the transdifferentiation of activated CD4^+^ lymphocytes into alloantigen-specific Treg cells (ag-Tregs) was further evaluated using Nanostring gene expression profiling, flow cytometry, ELISA, and in vivo migration assays. The results showed that the addition of the AS2863619 compound along with IL-2 generated effector memory ag-Tregs exhibiting tolerogenic activity, migration properties, and mechanisms for regulating immune homeostasis in the spleen. In conclusion, these findings suggest that the AS2863619-derived effector memory Tregs possess functional properties that support immune tolerance and regulate homeostasis in the spleen, thereby regulating the affinity of naïve T cells to alloantigens, highlighting their potential relevance in transplantology.

## 1. Introduction

Foxp3+ Regulatory T (Tregs) cells represent a specialized subset of CD4^+^ T lymphocytes with a wide range of immunoregulatory functions across various physiological and pathological contexts [1,2]. The transcription factor Forkhead box P3 (Foxp3) serves as the master regulator that controls the signaling pathways of Treg differentiation, especially those with high-affinity T-cell receptors (TCRs). The expression of Foxp3 triggers a shift in transcription factors from NFAT, AP-1, and NF-κB towards AML1/Runx1, leading to the stabilization of the Treg phenotype [3,4]. Recent studies have demonstrated that, upon expression of Foxp3 and key signaling mediators such as ENOLASE-1 and SCFAs, T-helper and T-effector cells can transdifferentiate into Tregs with suppressive properties, contributing to the maintenance of immune tolerance [5,6,7]. Thus, by utilizing the inherent regulatory potential of Tregs, it is theoretically possible to achieve suppression of inflammatory responses to a specific antigen without compromising the overall immune response.

**Figure 1:**
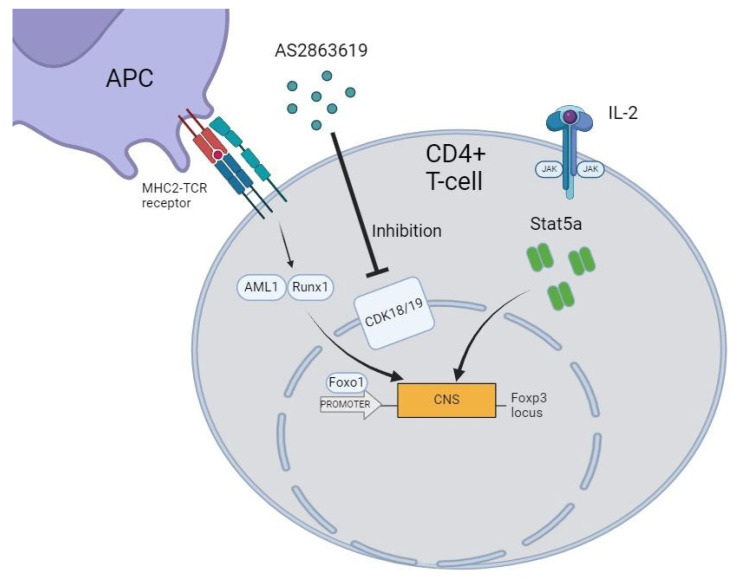
Principal of Treg induction using AS2863619.

Various approaches have been used to generate stable Foxp3+ Tregs, both in vivo and in vitro, that can suppress inflammatory responses of various etiologies, including autoimmune and transplantation reactions. For example, to address the issue of immune tolerance, a study was conducted to evaluate the effect of inhibiting cyclin-dependent kinase 8/19 (CDK8/19), key regulators of transcriptional signaling pathways, on the formation of the Treg pool in vitro (Figure 1). Pharmacological inhibitors of CDK8/19 have been shown to induce Foxp3 expression in both naive and activated CD4 lymphocytes [8]. Furthermore, it was found that CDK8/19 inhibitors, such as AS2863619 (4-[1-(2-methyl-1H-benzimidazol-6-yl)-1H-imidazo[4,5-c]pyridin-2-yl]-1,2,5-oxadiazol-3-amine, dihydrochloride), CCT251921 (8-(2-amino-3-chloro-5-(1-methyl-1H-indazol-5-yl)pyridin-4-yl)-2,8-diazaspiro[4.5]decan-1-one), and Senexin A, when used to induce Foxp3+ expression in Tregs in vitro, act synergistically with transforming growth factor beta 1 (TGF-β1) and help Tregs maintain their suppressive activity even in the presence of proinflammatory cytokines (Interleukin-6, Interleukin-12, and Interferon gamma). These inhibitors also enhance the expression of some Treg-specific genes (*Foxp3*, *Ctla4*, *Tnfrsf18*, and *Il2ra*), without triggering Treg-specific DNA demethylation. Functionally, Foxp3+ Tregs obtained using CDK8/19 inhibitors have shown the ability to suppress immune reactions in vitro as well as delayed-type hypersensitivity reactions and experimental autoimmune encephalomyelitis in mice in vivo [9].

**Figure 2:**
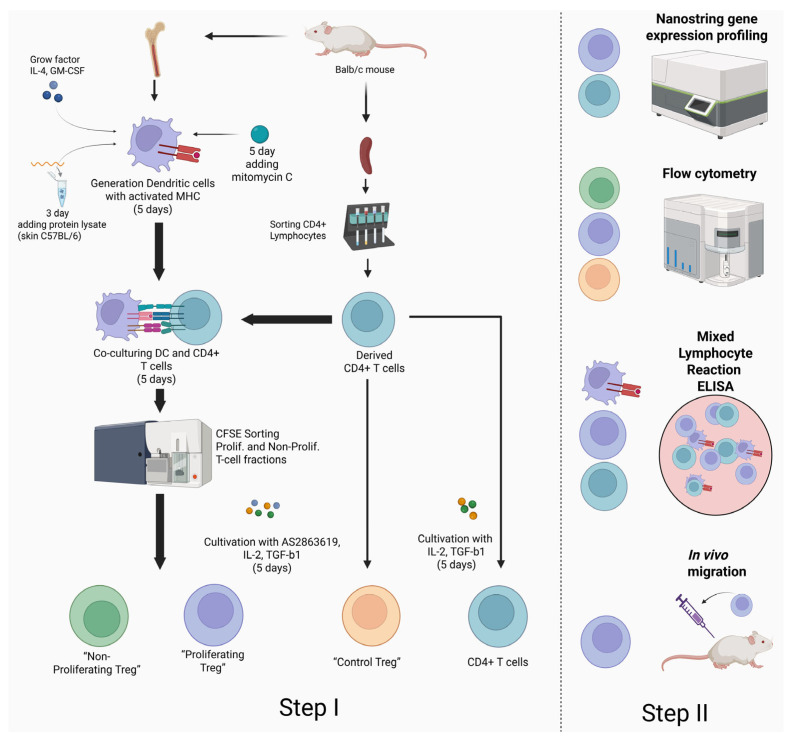
General scheme of experiment. Step I—The process of induction of Ag-Treg and control groups by the AS2863619 compound. Step II—Applied methods for assessing the obtained Ag-Treg by the AS2863619 compound.

Our study aims to obtain murine alloantigen-specific T-regulatory cells (ag-Tregs) with suppressor properties for the induction of immunological tolerance to foreign antigens. The derived ag-Tregs are expected to play a critical role in establishing a sustained antigen-specific immunosuppression, thereby providing a promising strategy for achieving stable immunosuppression for mitigating undesired graft-versus-host reactions and pathological autoimmune responses (Figure 2).

## 2. Results

### 2.1. NanoString Gene Expression Analysis Confirms the Transdifferentiation of CD4^+^ T Cells into CD4^+^ Regulatory T Cells

**Figure 3:**
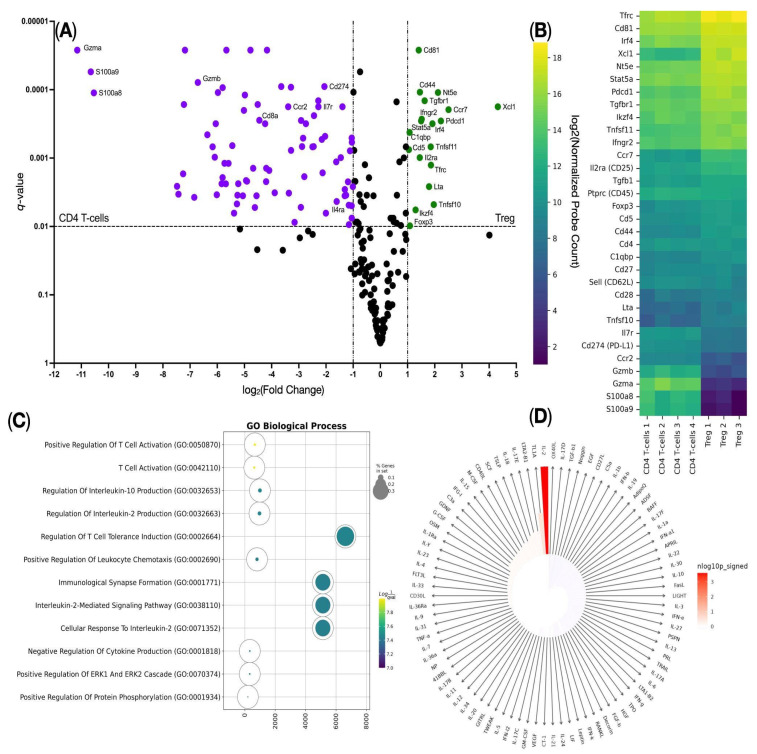
NanoString gene expression analysis of the CD4 T cells transdifferentiated into CD4 Treg. (**A**) Volcano plot of the differentially expressed genes: up-regulated genes are highlighted in green, down-regulated genes are highlighted in purple. (**B**) Heat map of the log2(Normalized Probe Counts) of the differentially expressed genes and the Treg-defining genes: the deep purple color marks the lowest marker expression, where the yellow color marks the highest marker expression. (**C**) Gene Ontology Biological Process overrepresentation analysis of the up-regulated genes: the yellow color corresponds to the lowest *q*-value, the deep purple color corresponds to the highest *q*-value, and the dot size reflects the percentage of genes included in the analysis relative to the full set of genes in the Gene Ontology Biological Process database. (**D**) Immune Response Enrichment Analysis (IREA) of the up-regulated genes: the cytokine that is significantly active in the analyzed Treg (IL-2) is highlighted in the deep red color (*n* = 1 technical replicate from 3 to 4 biological replicates in each group).

To confirm that the cocktail of the AS2863619 compound and the cytokines interleukin-2 (IL-2) and TGFB1 leads to the transdifferentiation of CD4^+^ T cells into CD4^+^ Tregs, we analyzed the gene expression of CD4^+^ T cells before and after adding the cocktail on the NanoString platform using the Mouse panel Immunology V1. We observed an increase in the expression of the genes *Cd81*, *Cd44*, *Tgbfr1*, *Nt5e*, *Ccr7*, *Xcl1*, *Ifngr2*, *Stat5a*, *Irf4*, *C1qbp*, *Cd5*, *Tnfsf11*, *Il2ra*, *Tfrc*, *Lta*, *Tnfsf10*, *Ikzf4*, and *Foxp3* (Figure 3A), among which the *Cd4*, *Il2ra* (CD25), and Foxp3 genes are canonical Treg markers. We also detected a high copy number of the Tfrc (CD71) gene, which indicates that the resulting Tregs were also activated (Figure 3A,B). We then performed Gene Ontology Biological Process overrepresentation analysis (GOOA) and Immune Response Enrichment Analysis (IREA) on overexpressed genes and found that such genes were overrepresented in Gene Ontology Biological Process terms such as “Regulation Of T-cell Tolerance Induction”, which also confirms the successful transdifferentiation of CD4^+^ T cells into CD4^+^ Tregs, and the “Cellular Response To Interleukin-2” term in the GOOA assay (Figure 3C), indicating the significant impact of IL-2 on the studied cells. This was further established in the IREA assay (Figure 3D). This suggests that the addition of the compound AS2863619 and the cytokine IL-2 leads to the expression of genes characteristic of the Treg population.

### 2.2. The Proportion of CD4^+^ CD44^+^ Regulatory T Cells Increases in Alloantigen-Specific Tregs After Co-Cultivation with DCs

**Figure 4:**
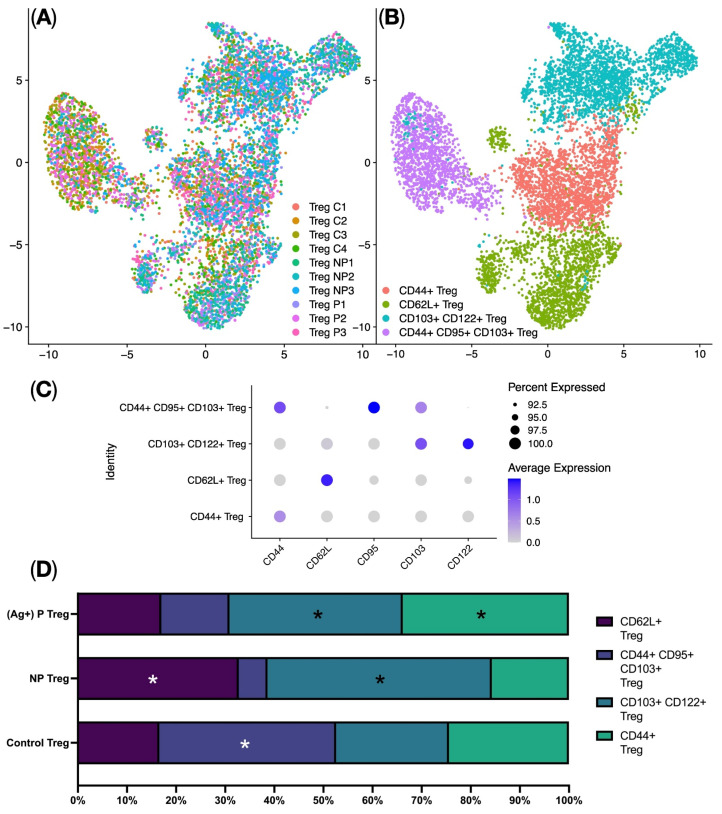
Flow cytometry single-cell data analysis. (**A**) UMAP plot of the Harmony data integration and batch correction. C—Control Treg; NP—Non-Proliferating Treg; P—Proliferating Treg (ag-Treg); (**B**) UMAP plot of the Treg-identified clusters. (**C**) Dot plot of marker expression per cluster: protein marker expression was Z-score-standardized, the gray color marks the lowest expression, while the deep blue color marks the highest expression, and the dot size represents the percentage of cells positive for the marker. White asterisks indicate significant differences in CD62L^+^ Treg and CD44^+^ CD95^+^ CD103^+^ Treg between the compared groups; Black asterisks indicate differences in CD103^+^ CD122^+^ Treg and CD44^+^ Treg between the compared groups. (**D**) (*n* = 1 technical replicates from 4 biological replicates in each group). Dot plot of the percentages of the clusters in each biogroup: horizontal lines on the plot depict medians.

The analysis of Treg subpopulations was performed using the Seurat single-cell analysis library. This involved Centered Log-ratio (CLR) data normalization, data integration using Harmony, UMAP (Uniform Manifold Approximation and Projection) dimensionality reduction (Figure 4A), and identification of clusters corresponding to biological Treg subpopulations. The analysis identified four clusters of regulatory T cells: CD44^+^ Tregs, CD62L^+^ Tregs, CD103^+^ CD122^+^ Tregs, and CD44^+^ CD95^+^ CD103^+^ Tregs (Figure 4B,C). Analyzing the proportions of the different Treg clusters among the studied groups revealed some significant changes. Specifically, a higher proportion of CD44^+^ Tregs was observed in the Proliferating Treg group (ag-Treg) compared to the other groups. Conversely, the Non-Proliferating Treg group demonstrated higher proportions of CD62L^+^ Tregs, CD103^+^ CD122^+^ Tregs, and CD44^+^ CD95^+^ CD103^+^ Tregs, along with a lower proportion of CD44^+^ Tregs compared to the other groups (see Figure 4D). Thus, we can say that when compound AS2863619 is added, effector memory Tregs are predominantly generated.

### 2.3. Addition of Ag-Tregs to a Mixed Lymphocyte Reaction Increases the Production of Cytokines IL-2 and IL-10

**Figure 5:**
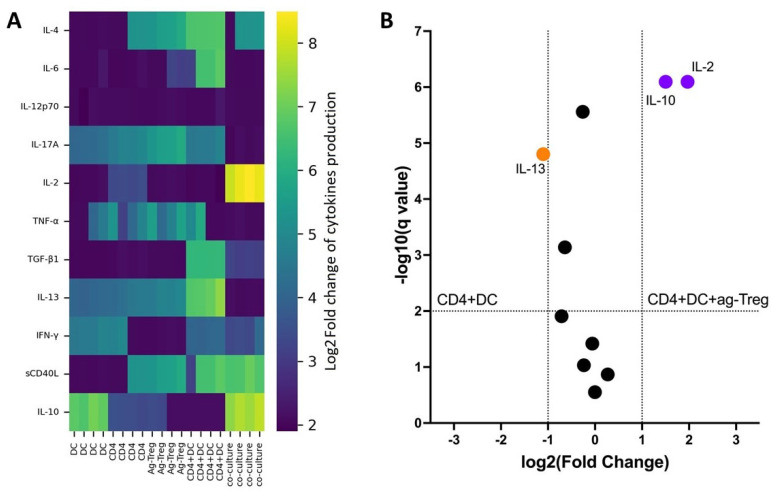
(**A**) Log2-transformed difference fold changes and (**B**) volcano plot in cytokine production in mixed lymphocyte reaction with added ag-Tregs obtained by AS2863619 compound (*n* = 1 technical replicate from 4 biological replicates in each group). Data are presented as a heat map and were pre-optimized by log2 transformation. Co-culture: A group of CD4 T cells to which dendritic cells and ag-Tregs were added. One-way ANOVA analysis (paired comparisons with the *q*-values < 0.01).

In vitro assessment of cytokine production in a mixed lymphocyte reaction demonstrated that, in cultures containing dendritic cells and CD4 T cells with the addition of ag-Tregs, a significantly higher concentration of IL-2 and IL-10 cytokines was observed compared to other groups (Figure 5A). Furthermore, the volcanic plot confirmed higher concentrations of IL-2 and IL-10 cytokines and lower concentrations of IL-13 in CD4 + DC + ag-Treg cultures compared to the CD4 + DC group (Figure 5B). These results further support the assertion about the effectiveness of ag-Tregs in the targeted induction of tolerance.

### 2.4. In Vivo Migration Assay Demonstrates a Characteristic Migratory Pattern of Ag-Tregs into the Spleen in a Balb/c Mouse Model

**Figure 6:**
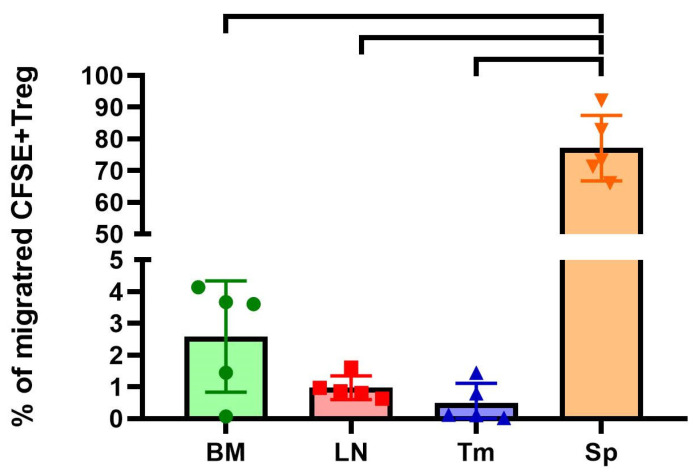
Distribution of CFSE+ Foxp3+ Tregs in Balb/c murine organs in vivo (*n* = 5). BM—bone marrow; LN—lymph nodes; Tm—thymus; Sp—spleen. One-way ANOVA analysis with Dunnett correction was applied (paired comparisons with the *q*-values < 0.001 are shown).

The final stage of our study was to evaluate the migration pattern of the obtained ag-Tregs in vivo in a mouse model using flow cytometry with the help of the fluorescence vital dye CFSE. The results showed that on day 2 after administration, approximately 3.1 million cells or 77.13 ± 8.27% (mean ± SEM) of the total number of injected CFSE+ ag-Tregs were located in the spleen (Figure 6) (*n* = 1 technical replicate from 5 biological replicates in each group).

**Figure 7:**
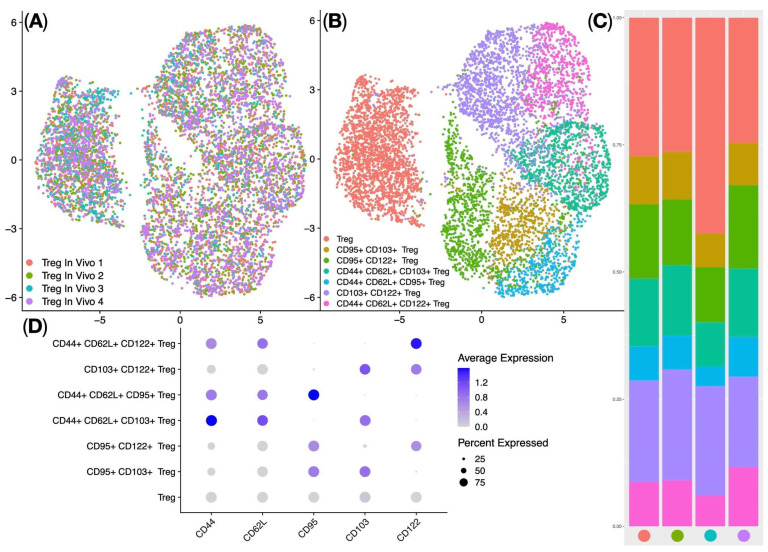
In Vivo Treg flow cytometry single-cell data analysis. (**A**) UMAP plot of the Harmony data integration and batch correction. (**B**) UMAP plot of the In Vivo Treg clusters. (**C**) Stacked bar plot of the proportions of clusters per sample: sample colors are in accordance with the colors of samples in subfigure (**A**), and cluster colors are in accordance with the colors of clusters in subfigure (**B**). (**D**) Dot plot of marker expression: protein marker expression was Z-score-standardized, the gray color marks the lowest expression, while the deep blue color marks the highest expression, and the dot size represents the percentage of cells positive for the marker (*n* = 1 technical replicate from 4 biological replicates in each group).

Treg subpopulations in the spleens of Balb/c mice (In Vivo Treg) were further analyzed using the Seurat single-cell analysis library: this involved Centered Log-ratio (CLR) data normalization, data integration using Harmony, UMAP (Uniform Manifold Approximation and Projection) dimensionality reduction (Figure 7A), and identification of clusters corresponding to biological Treg subpopulations. The analysis identified seven clusters of regulatory T cells—classical regulatory T cells (Treg), CD95^+^ CD103^+^ Treg, CD95^+^ CD122^+^ Treg, CD103^+^ CD122^+^ Treg, CD44^+^ CD62L^+^ CD103^+^ Treg, CD44^+^ CD62L^+^ CD95^+^ Treg, and CD44^+^ CD62L^+^ CD122^+^ Treg (Figure 7B,D)—which were dominated by classical regulatory T cells (median = 28%) (Figure 7C). In summary, ag-Tregs predominantly migrated to the spleen of mice, and when ag-Tregs were detected in the spleen, they had the phenotype of classical Tregs.

## 3. Discussion

In this study, we assessed the ability of the CDK8/19 inhibitor AS2863619 to generate stable alloantigen-specific regulatory T cells (ag-Tregs) in vitro. The resulting ag-Tregs were analyzed to assess their phenotypic and functional characteristics as a Treg population. The data obtained suggest that the AS2863619 compound may be useful in cellular technologies that aim to regulate immune reactions after transplantation.

Gene expression profiling of ag-Tregs derived from activated CD4^+^ lymphocytes showed up-regulated expression of key genes that are characteristic of the Treg phenotype (*Cd44*, *Tgbfr1*, *Stat5a*, *Il2ra*, *Ikzf4*, and *Foxp3*) (Figure 3A,B). Among these, the *Ikzf4* (Ikaros) and *Stat5a* genes are particularly noteworthy, as they play a pivotal role in switching the TCR signaling pathway during the transition of T cells towards Treg [10,11]. In addition, these transcription factors enhance the activity of the transcription factor FOXO1, which is critically important upon activation of Foxp3 expression [12]. The observed up-regulation in the expression of the *Cd71*, *Nt5e*, and *Xcl1* genes in ag-Tregs indicates the suppressive potential of the activated Tregs (Figure 3A,B). Notably, *Nt5e* has been shown to be involved in the transformation of ATP to the immunosuppressive adenosine, which enhances the expression of the CD39/CD73 receptors and the transcription factor HIF-1α, which ultimately leads to the formation of Tregs with suppressive capacity [13]. Furthermore, the increased expression of the C-C chemokine receptor 7 gene (*Ccr7*) is of particular importance, as it is extremely important for the migration of T lymphocytes. The migration of T lymphocytes to the peripheral organs of the immune system is carried out by two ligands, CCL19 and CCL21, that bind to the CCR7 receptor. These ligands are activated in cells that are in the periphery (for example, lymphatic endothelial cells) [14], which are present in various tissues of the body, such as the skin, where they produce chemotaxis molecules that enhance the expression of CCR7 and thereby induce migration of activated Tregs to the periphery to participate in the regulation of the immune response [15]. Gene Ontology Biological Process overrepresentation analysis of the up-regulated genes showed a predominance of genes associated with “Regulation Of T-cell Tolerance Induction”, which indicates the capability of the transdifferentiated Tregs to mediate tolerance (Figure 3C). Additionally, enrichment in “Cellular Response to Interleukin-2” further verifies the efficacy of using the AS2863619 compound in combination with IL-2 to generate Tregs in vitro [9]. Furthermore, the IREA of the up-regulated genes showed that the addition of IL-2 has a significant effect on the generation of ag-Tregs (Figure 3D), as it is known that IL-2 has a key role in promoting the differentiation of Tregs and cell survival in the microenvironment, even at low doses [16,17]. Given that the activation of the T-cell receptor was carried out by the direct alloreaction pathway mechanism and that the generation of Tregs using AS2863619 requires an activated TCR [9], it can be concluded that T cells are alloantigen-specific. This is also supported by previous studies conducted within the framework of clonotype studies, which obtained data on the various mechanisms of activation of the TCR repertoire in vitro and in vivo [18]. Collectively, these findings indicate that the addition of IL-2 and the AS2863619 compound upon stimulation of the TCR of CD4^+^ lymphocytes promotes Treg differentiation in vitro, with the resulting Tregs having suppressive properties that allow for the induction of tolerance in the periphery.

Studying the subpopulations of Tregs in the Control, Proliferating (ag-Tregs), and Non-Proliferating Treg groups showed that when the AS2863619 compound is added to CD4^+^ splenocytes, Tregs are formed with pronounced features of effector memory Tregs, as evidenced by the expression of CD44^+^, CD62L^+^, and CD103^+^ (Figure 4A,B). It is well-established that upon strong TCR stimulation of T lymphocytes, the AS2863619 compound phosphorylates the tyrosine in the C-terminal domain of the TCR, leading to the maintenance of STAT5a activity [9]. STAT5a subsequently binds to a conserved noncoding region (CNS2), which supports the expression of Foxp3, contributing to the formation of a full-fledged Treg phenotype [19]. The increase in the expression of the *Il2ra*, *Tnfrsf18*, *Foxp3*, *Ccr4*, and *Ikzf4* genes (Figure 3A), together with the data obtained from flow cytometry, strengthens the assumption that it is effector memory Tregs that are formed in the analyzed groups of ag-Tregs (Figure 4C,D). This is consistent with previously obtained data on the study of the properties of ag-Tregs to regulate lymphocyte proliferation, where it was found that induced ag-Tregs suppress the proliferation of CD4^+^ lymphocytes to alloantigens in mixed lymphocyte reaction [20]. In connection with this, we can say that Tregs obtained using AS2863619 have suppressor properties. There is limited research on effector memory Treg cells; it is known that they are localized in the skin and in secondary lymphoid organs, produce large amounts of IFN-γ, and are powerful inhibitors of inflammatory reactions against self-antigens, and this may be an important factor in maintaining tissue homeostasis after transplantation [21].

In conclusion, the investigation into the mixed lymphocyte reaction revealed that the incorporation of ag-Tregs into the dendritic cell and CD4 culture enhanced the production of IL-2 and IL-10 cytokines (see Figure 5). It is well established that Tregs have the capacity to induce the differentiation of naive T cells into peripheral Tregs by modulating the microenvironment [22]. Furthermore, this process can be alloantigen-specific, and upon stimulation of T-cell receptors by IL-2, favorable conditions are created for T cells [23]. It has been established that IL-10 production by Tregs can suppress the immune response [24]. In consideration of the data that has previously been presented, it can be concluded that ag-Tregs obtained using AS2863619 can create conditions for the induction of Tregs and immunological tolerance in general, a hypothesis that is further supported by the in vivo migration data. When assessing the distribution of injected ag-Tregs into various mouse organs, we observed targeted migration of Tregs into the spleens of Balb/c mice (Figure 4). Additionally, analysis of CFSE+ Foxp3+ Tregs revealed seven populations of migrated Tregs in the spleen of mice (Figure 7A,B) that exhibit features of effector memory Tregs [25,26]. The migration of effector memory Tregs to the spleen suggests adoptive transfer of CD62L^+^ CD103^+^ CCR7^+^ ag-Tregs from lymph nodes to the spleen to modulate the tolerance in naïve T cells. For example, in the process of modulating tolerance to alloantigens, Tregs can migrate from lymph nodes into the spleen (production of IL-10, TGF-β1, etc.) to ensure effective regulation of the host reaction of trained T cells during allograft [27,28].

Based on the above-mentioned results, we can conclude that the pharmacological compound AS2863619 promotes the transdifferentiation of activated CD4^+^ T cells into Tregs with suppressor properties and a characteristic migratory pattern into the spleen to regulate various immune responses in vitro and in vivo.

## 4. Materials and Methods

### 4.1. Laboratory Animals

The study used 9 male mice from line C57BL/6 and 27 male mice from line BALB/c, aged 12 weeks. The mice were housed under normal vivarium conditions without exposure to pathogens, with unlimited access to water and food while maintaining a natural dark/light cycle. The conditions for keeping the mice, conducting the experiment, humane treatment, and anesthesia when removing the animal from the experiment were approved by the local committee of the RIFCI.

### 4.2. Deriving Dendritic Cells

Dendritic cells (DCs) were derived from red bone marrow cells. For this purpose, 3 × 10^6^ bone marrow cells, isolated according to the standard method from femurs [29], were cultured in 6-well plates (TPP, Trasadingen, Switzerland) in 3 mL of an RPMI-1640 medium supplemented with 10% fetal bovine serum (FCS) (HyClone, Logan, UT, USA), 2 mM L-glutamine (Biolot, St. Petersburg, Russia), 5 × 10^−5^ mM mercaptoethanol (Sigma, St. Louis, MA, USA), 25 mM HEPES (Biolot, Russia), 80 μg/mL gentamicin (KRKA, Novo Mesto, Slovenia), 100 μg/mL ampicillin (Sintez, Kurgan, Russia) (will be further referred to as full-media RPMI-1640) with the addition of 100 ng/mL GM-CSF and IL-4 (Biolegend, San Diego, CA, USA) within 5 days. On day 3, the medium was changed, and growth factors were added. On day 5, the dendritic cell content in the culture was assessed using flow cytometry (90% of the population generated cDC2s). The resulting dendritic cells were treated with 25 μg/mL of the cytostatic drug mitomycin-C for 30 min at 37 °C in a CO_2_ incubator. After culturing with mitomycin-C, the cells were washed twice with RPMI 1640. Treated DCs were seeded into a 6-well plate (TPP, Switzerland) at 1 × 10^6^ cells per well in 1 mL of full-media RPMI-1640.

### 4.3. Generation of Ag-Specific Tregs from Effector CD4^+^ T Lymphocytes

Alloantigen-specific T-regulatory cells were obtained from the CD4^+^ fraction of splenocytes. To achieve this, at the first stage, splenocytes were labeled with the vital dye CFSE (5 mmol/mL) for 20 min according to the manufacturer’s instructions (Biolegend, San Diego, CA, USA). Then, the CFSE+ CD4^+^ lymphocytes were magnetically sorted according to the manufacturer’s protocol (Mojosort, San Diego, CA, USA). The sorting purity was 98–99%. CFSE+ CD4^+^ lymphocytes were co-cultured with mitomycin-treated DCs in a ratio of 1:5 for 5 days. Half of the medium was changed on the 3rd day of cultivation. After that, the proliferating and non-proliferating fractions of CFSE+ CD4^+^ lymphocytes were isolated using BD FACSAria™ I, a flow cytometer with sorting function. The proliferating fraction of CFSE+ CD4^+^ lymphocytes was divided into two parts; one of them was used as a control in the NanoString experiment, where the total RNA was isolated (“CD4 T-cells” in the NanoString data). The second part, together with the non-proliferating fraction of CFSE+ CD4^+^ lymphocytes, was placed in 24-well plates (TPP, Trasadingen, Switzerland) at a concentration of 1 million/mL in full-media RPMI-1640 with the addition of 1 mm/mL of the AS2863619 compound (SelleckChem, Houston, TX, USA), 300,000 U/mL IL-2 (Biolegend, San Diego, CA, USA), and 2.5 μg/mL TGF-β1 (Elabscience, Houston, TX, USA) for the transdifferentiation of allospecific T lymphocytes into Tregs. Cultivation was carried out for 5 days, followed by assessment of the proportions of Treg subpopulations (“Proliferating Treg” and “Non-Proliferating Treg” in the flow cytometry data) and isolation of total RNA from the proliferating fraction of Tregs (“Treg” in the NanoString data). As a control, we used a population of CD4^+^ T lymphocytes cultured with the AS2863619 compound in the absence of DCs to obtain ag-nonspecific Tregs (“Control Treg” in the flow cytometry data).

### 4.4. Total RNA Extraction

We isolated the total RNA from 200,000 cells with the Total RNA Purification Kit (Cat. 17250, Norgen Biotek, Thorold, Ont, Canada) according to the manufacturer’s instructions. We measured the concentration of the total RNA in each sample on a Qubit 4 (Thermo Fisher Scientific, Waltham, MA, USA) using the Qubit™ RNA High Sensitivity (HS) kit (Thermo Fisher Scientific, Waltham, MA, USA). The total RNA samples were frozen at −80 °C until NanoString gene expression profiling.

### 4.5. Nanostring Gene Expression Profiling

To determine the change in the T-cell transcriptome with the addition of AS2863619 to the Nanostring, we compared the resulting Treg and CD4 T cells without the addition of AS2863619. We performed gene expression profiling with the help of the Nanostring nCounter SPRINT Profiler analytical system using 100 ng of total RNA from each sample (*n* = 4) and the nCounter Mouse Immunology v1 panel as described previously [30]. We performed normalization and QC in nSolver 4 using added synthetic positive controls and the 14 housekeeping genes included in the panel. Samples that did not meet the quality control requirements specified in the User Manual of the nSolver 4.0 analysis software were excluded from the analysis. We then performed background thresholding on the normalized data to remove non-expressing genes. The background level was determined as the mean of the POS_D controls, and the genes that were below the background level in at least one sample were removed.

### 4.6. Flow Cytometry

**Scheme 1:**
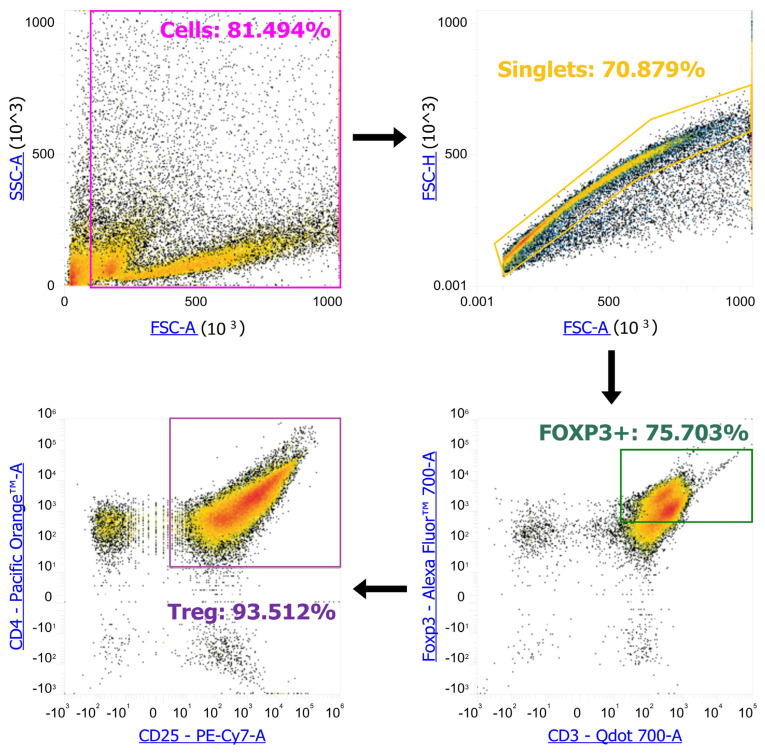
Flow cytometry gating of regulatory T cells.

To understand how the subpopulation composition of Tregs changes when AC286319 is added to proliferating activated T cells, we used “Non-Proliferating Tregs” and “Control Tregs” as controls. To determine different types of Treg populations, we used anti-CD3-Bv711 #100241, anti-CD4-Bv605 #100547, anti-CD25-PeCy7 #101916, anti-CD44-APC-Cy7 #103028, anti-CD62L-PerCP #103028, anti-CD95-APC #152604, anti-CD122-PE #105906, anti-CD69-Bv421 #104528, anti-CD103-AF488 #121408, and anti-FoxP3-PE #126422 (Biolegend, San Diego, CA, USA) antibodies. The staining was performed according to the manufacturer’s standard protocol. The analysis was performed on an Attune NxT flow cytometer (Life Technologies, Waltham, MA, USA). We gated cells from all events, singlets from cells, FOXP3+ cells from singlets, and CD4^+^ CD25+ cells from FOXP3+ cells to obtain Treg (Scheme 1). We then exported gated Treg events as .fcs files and converted them into .csv files via fcsfarser.

### 4.7. Flow Cytometry Single-Cell Data Analysis

We imported .csv flow cytometry data files into Seurat V5 [31], performed data quality control (nCountAb < 1,000,000), data normalization via Centered Log-ratio (CLR), Principal Component Analysis (PCA) dimensionality reduction, data batch correction and integration via Harmony [32], Uniform Manifold Approximation and Projection (UMAP) dimensionality reduction using 7 Harmony-corrected Principal Components, and found neighbors and found clusters. We identified clusters using *FeaturePlot* of marker expression and agglomerated and renamed clusters to match biological Treg subpopulations. We then created Harmony integration *DimPlot*, Cluster *DimPlot*, *DotPlot* of marker expression and either created a stacked bar plot using ggplot2, or exported cell % per sample per cluster to GraphPad Prism 10.2.1 (GraphPad Software, San Diego, CA, USA), where we performed Kolmogorov–Smirnov normality test and created a QQ-plot to test the data for normality, performed two-way ANOVA with Tukey correction, and created a dot plot of the imported data.

### 4.8. Differential Gene Expression, GO BP Overrepresentation Analysis, and Immune Response Enrichment Analysis

We log2-transformed the NanoString data (NanoString data distribution is log-normal) and performed differential gene expression analysis using multiple T-tests (we considered *q*-values < 0.01 and log2(fold change) > 1.0 or log2(fold change) < −1.0 to be significant) and created volcano plots in GraphPad Prism 9.4.1 We then created a heat map of the differentially expressed and Treg-defining genes via bioinfokit [33] and performed Gene Ontology Biological Process overrepresentation analysis of the up-regulated differentially expressed genes via GSEApy [34] and Immune Response Enrichment Analysis via IREA using a Treg preset [35].

### 4.9. Mixed Lymphocyte Reaction (MLR)

Mixed lymphocyte reaction (MLR) was performed as follows: First, dendritic cells of the C57BL/6 line and Ag-Treg CD4 line balb/c were induced. To determine which cytokines are produced by the AS2863619-derived Ag-Tregs to suppress T-cell proliferation, the following analysis groups were selected: a culture containing only dendritic cells, CD4, or Ag-Treg (level of cytokine production in monoculture); a group where only DC or Ag-Treg were added to CD4; and finally, the group of interest is CD4 T cells with the addition of DC and Ag-Treg. Co-cultivation was carried out for 5 days, with the collection of conditioned media for ELISA.

### 4.10. Analysis of Ag-Treg Co-Culture Media Using ELISA

Four microliters of each of the samples of conditioned media from DC, CD4, ag-Tregs, and co-culture (DC + Ag-Treg + CD4) were prepared for a cytokine quantification (IL-4, IL-6, IL-12p70, IL-2, TNF-α, TGF-β1, IL-13, IFN-γ, sCD40L, and IL-10) with a LegendPlex Mouse B cell panel—S/p (12-plex)—with Filter Plate assay (Cat.№ 740827, Biolegend, San Diego, CA, USA) according to the manufacturer’s recommendations and analyzed on the BioLegend’s LEGENDplex™ data analysis software version 8. ELISA analysis was performed using a Varioskan LUX Multimode Microplate Reader (Thermo Fisher Scientific, Waltham, MA, USA).

### 4.11. Assessing the Migratory Potential of Ag-Tregs In Vivo

To assess the in vivo migration, 4 × 10^6^ ag-Tregs of Balb/c mice were stained with the vital dye CFSE (5 mMol/mL) and injected into the tail vein of Balb/c mice. On the second day after injection of CFSE+ ag-Tregs, mice were sacrificed by cervical dislocation, and the bone marrow, thymus, spleen, axillary, and inguinal lymph nodes were harvested. To study the migratory potential of ag-Tregs, we selected organs of interest based on the obtained data on the phenotype and functional properties of Tregs, as well as on the basis of data on the migration of activated Tregs [36,37]. The harvested organs were dissected into small pieces and then homogenized using a magnetic stirrer in RPMI-1640 for 30 min. The organ tissue fragments and cell suspension were mixed in an RPMI-1640 medium containing 2 mg/mL collagenase (Biolot, Russia) for 30 min using a magnetic stirrer. The obtained cell suspension from each organ was then rinsed with an FCS-containing medium, filtered through a 45 μm nylon filter, and used to assess in vivo migration of ag-Tregs with flow cytometry. Ag-Tregs were gated as shown in Scheme 1 (“Treg In Vivo” in the flow cytometry data).

### 4.12. Statistical Analysis of Other Data

Statistical data processing was performed using GraphPad Prism 9.4.1/10.2.1 software (GraphPad Software, USA). The obtained data were checked for normality using the Kolmogorov–Smirnov and Shapiro–Wilk tests. The data were then analyzed using appropriate statistical methods. The statistical methods used are indicated in the figure legends.

## 5. Conclusions

The addition of the AS2863619 compound and IL-2 to a culture of activated CD4^+^ lymphocytes stimulates the generation of proliferating alloantigen-specific Tregs. At the same time, the resulting ag-Tregs were found to express genes that are characteristic of effector memory Tregs and chemokine receptor genes that directed migration into the spleens in murine models, which makes it important to study their ability to induce immunological tolerance and suppress the immune response. Also, studying the effector memory potential of ag-Tregs in various functional tests will provide additional information about the properties of regulatory T cells in vitro and in vivo. Finally, investigations centered on studying the interaction of Foxp3 with the CDK8/19-STAT5 signal upon activation of the TCR will provide therapeutic benefits that can be applied in experimental transplantology.

## Data Availability

The data that support the findings of this study are available upon an email request from the corresponding author, S.V. Sennikov.

## References

[1] Kim J.H., Kim B.S., Lee S.K. (2020). Regulatory T Cells in Tumor Microenvironment and Approach for Anticancer Immunotherapy. Immune Netw..

[2] Pilat N., Sprent J. (2021). Treg Therapies Revisited: Tolerance Beyond Deletion. Front. Immunol..

[3] Raugh A., Allard D., Bettini M. (2022). Nature vs. nurture: FOXP3, genetics, and tissue environment shape Treg function. Front Immunol..

[4] Vent-Schmidt J., Han J.M., MacDonald K.G., Levings M.K. (2014). The role of FOXP3 in regulating immune responses. Int. Rev. Immunol..

[5] Kurniawan H., Soriano-Baguet L., Brenner D. (2020). Regulatory T cell metabolism at the intersection between autoimmune diseases and cancer. Eur. J. Immunol..

[6] Howie D., Ten Bokum A., Necu A.S., Cobbold S.P., Waldmann H. (2018). The Role of Lipid Metabolism in T Lymphocyte Differentiation and Survival. Front. Immunol..

[7] Li Y., Lu Y., Wang S., Han Z., Zhu F., Ni Y., Liang R., Zhang Y., Leng Q., Wei G. (2016). USP21 prevents the generation of T-helper-1-like Treg cells. Nat. Commun..

[8] Guo Z., Wang G., Lv Y., Wan Y.Y., Zheng J. (2019). Inhibition of Cdk8/Cdk19 Activity Promotes Treg Cell Differentiation and Suppresses Autoimmune Diseases. Front. Immunol..

[9] Akamatsu M., Mikami N., Ohkura N., Kawakami R., Kitagawa Y., Sugimoto A., Hirota K., Nakamura N., Ujihara S., Kurosaki T. (2019). Conversion of antigen-specific effector/memory T cells into Foxp3-expressing Treg cells by inhibition of CDK8/19. Sci. Immunol..

[10] Polak K., Marchal P., Taroni C., Ebel C., Kirstetter P., Kastner P., Chan S. (2023). CD4+ regulatory T cells lacking Helios and Eos. Biochem. Biophys. Res. Commun..

[11] Wei L., Laurence A., O’Shea J.J. (2008). New insights into the roles of Stat5a/b and Stat3 in T cell development and differentiation. Semin. Cell Dev. Biol..

[12] Georgiev P., Charbonnier L.M., Chatila T.A. (2019). Regulatory T Cells: The Many Faces of Foxp3. J. Clin. Immunol..

[13] Jarvis L.B., Rainbow D.B., Coppard V., Howlett S.K., Georgieva Z., Davies J.L., Kaur Mullay H., Hester J., Ashmore T., Van Den Bosch A. (2021). Therapeutically expanded human regulatory T-cells are super-suppressive due to HIF1A induced expression of CD73. Commun. Biol..

[14] Chauhan S.K., Saban D.R., Dohlman T.H., Dana R. (2014). CCL-21 conditioned regulatory T cells induce allotolerance through enhanced homing to lymphoid tissue. J. Immunol..

[15] Sharma A., Rudra D. (2018). Emerging Functions of Regulatory T Cells in Tissue Homeostasis. Front Immunol..

[16] Harris F., Berdugo Y.A., Tree T. (2023). IL-2-based approaches to Treg enhancement. Clin. Exp. Immunol..

[17] Fan M.Y., Low J.S., Tanimine N., Finn K.K., Priyadharshini B., Germana S.K., Kaech S.M., Turka L.A. (2018). Differential Roles of IL-2 Signaling in Developing versus Mature Tregs. Cell Rep..

[18] Tereshchenko V., Shevyrev D., Fisher M., Bulygin A., Khantakova J., Sennikov S. (2023). TCR Sequencing in Mouse Models of Allorecognition Unveils the Features of Directly and Indirectly Activated Clonotypes. Int. J. Mol. Sci..

[19] Kawakami R., Kitagawa Y., Chen K.Y., Arai M., Ohara D., Nakamura Y., Yasuda K., Osaki M., Mikami N., Lareau C.A. (2021). Distinct Foxp3 enhancer elements coordinate development, maintenance, and function of regulatory T cells. Immunity.

[20] Bulygin A.S., Khantakova J.N., Fisher M.S., Tereshchenko V.P., Kurilin V.V., Shkaruba N.S., Sennikov S.V. (2023). Generation of stable antigen-specific T-regulatory cells using blockers of cyclin-dependent protein kinases. Immunology.

[21] Cheru N., Hafler D.A., Sumida T.S. (2023). Regulatory T cells in peripheral tissue tolerance and diseases. Front. Immunol..

[22] Goswami T.K., Singh M., Dhawan M., Mitra S., Emran T.B., Rabaan A.A., Mutair A.A., Alawi Z.A., Alhumaid S., Dhama K. (2022). Regulatory T cells (Tregs) and their therapeutic potential against autoimmune disorders—Advances and challenges. Hum. Vaccin. Immunother..

[23] Abbas A.K. (2020). The Surprising Story of IL-2: From Experimental Models to Clinical Application. Am. J. Pathol..

[24] Saraiva M., O’Garra A. (2010). The regulation of IL-10 production by immune cells. Nat. Rev. Immunol..

[25] Shevyrev D., Tereshchenko V. (2020). Treg Heterogeneity, Function, and Homeostasis. Front. Immunol..

[26] Khantakova J.N., Bulygin A.S., Sennikov S.V. (2022). The Regulatory-T-Cell Memory Phenotype: What We Know. Cells..

[27] Wei S., Kryczek I., Zou W. (2006). Regulatory T-cell compartmentalization and trafficking. Blood..

[28] Milanez-Almeida P., Meyer-Hermann M., Toker A., Khailaie S., Huehn J. (2015). Foxp3+ regulatory T-cell homeostasis quantitatively differs in murine peripheral lymph nodes and spleen. Eur. J. Immunol..

[29] Liu X., Quan N. (2015). Immune Cell Isolation from Mouse Femur Bone Marrow. Bio Protoc..

[30] Perik-Zavodskaia O., Perik-Zavodskii R., Nazarov K., Volynets M., Alrhmoun S., Shevchenko J., Sennikov S. (2023). Murine Bone Marrow Erythroid Cells Have Two Branches of Differentiation Defined by the Presence of CD45 and a Different Immune Transcriptome Than Fetal Liver Erythroid Cells. Int. J. Mol. Sci..

[31] Hao Y., Stuart T., Kowalski M.H., Choudhary S., Hoffman P., Hartman A., Srivastava A., Molla G., Madad S., Fernandez-Granda C. (2023). Dictionary Learning for Integrative, Multimodal and Scalable Single-Cell Analysis. Nat. Biotechnol..

[32] Korsunsky I., Millard N., Fan J., Slowikowski K., Zhang F., Wei K., Baglaenko Y., Brenner M., Loh P., Raychaudhuri S. (2019). Fast, sensitive and accurate integration of single-cell data with Harmony. Nat. Methods.

[33] Bedre R. (2022). Bioinfokit: Bioinformatics Data Analysis and Visualization Toolkit.

[34] Fang Z., Liu X., Peltz G. (2023). GSEApy: A comprehensive package for performing gene set enrichment analysis in Python. Bioinformatics.

[35] Cui A., Huang T., Li S., Ma A., Pérez J.L., Sander C., Keskin D.B., Wu C.J., Fraenkel E., Hacohen N. (2024). Dictionary of immune responses to cytokines at single-cell resolution. Nature.

[36] Chen W., Cao Y., Zhong Y., Sun J., Dong J. (2022). The Mechanisms of Effector Th Cell Responses Contribute to Treg Cell Function: New Insights into Pathogenesis and Therapy of Asthma. Front Immunol..

[37] Miragaia R.J., Gomes T., Chomka A., Jardine L., Riedel A., Hegazy A.N., Whibley N., Tucci A., Chen X., Lindeman I. (2019). Single-Cell Transcriptomics of Regulatory T Cells Reveals Trajectories of Tissue Adaptation. Immunity.

[38] (2010). Guide for the Care and Use of Laboratory Animals.

